# Acute Idiopathic Blind Spot Enlargement Syndrome—New Perspectives in the OCT Era

**DOI:** 10.3390/jcm11185278

**Published:** 2022-09-07

**Authors:** Julian A. Zimmermann, Nicole Eter, Julia Biermann

**Affiliations:** Department of Ophthalmology, University of Muenster Medical Center, Albert-Schweitzer-Campus 1, 48149 Muenster, Germany

**Keywords:** blind spot enlargement, optical coherence tomography, retinopathy, optic neuritis, peripapillary, optic disc, visual field defect, young adults

## Abstract

Acute idiopathic blind spot enlargement syndrome (AIBSES) is characterized by unilateral visual field loss in the blind spot area, acute onset photopsia, and funduscopically few or no optic disc changes. AIBSES predominantly affects young adults and is often misdiagnosed as optic neuritis because of low awareness. Optical coherence tomography (OCT) has become the gold standard in diagnosing AIBSES as a disease of the outer retina. In our case series, we present three consecutive patients with AIBSES followed prospectively with and without steroid therapy. The patients, aged 25 to 27 years, presented in our neuroophthalmology department between 2020 and 2021. We report their disease course and management and discuss therapeutic options, as no well-established procedures exist. Common pitfalls and diagnostic errors are analysed. Two women and one man showed unilateral acute-onset photopsia and blind spot enlargement on perimetry without visual acuity reduction. Spectral domain OCT (Heidelberg Engineering, Heidelberg, Germany) revealed marked peripapillary changes in the ellipsoid zone and autofluorescence in all patients, corresponding to faint blurring of the optic disc margin. Characteristically, there was no P100 latency delay in the visual evoked potential in any of the patients. Two patients received weight-adapted oral prednisolone, which was gradually tapered over six to eight weeks. Two patients showed full recovery of their symptoms at six and seven months after onset, while mild defect healing was seen in one treated patient after 12 months. Follow-up OCT showed restoration of the outer retinal layers 6–12 months after disease onset. Careful history taking and an unprejudiced ophthalmological workup helps in diagnosing AIBSES in young adults with unilateral acute visual field defects. While its etiology is still unclear, accurate diagnosis of AIBSES can be made with peripapillary OCT. In our cases, the disease course of AIBSES was much better than its reputation. Early corticosteroid treatment may support outer retinal reorganisation, which can be followed with OCT in accordance with visual field restoration. This should be addressed in a prospective study.

## 1. Introduction

In 1988, Fletcher et al., were the first to report acute idiopathic blind spot enlargement syndrome (AIBSES), characterised by acute-onset monocular photopsia and temporal scotoma, corresponding with blind spot enlargement in visual field testing and no or mild funduscopic changes of the retina and the optic nerve head [1]. To our knowledge, fewer than 100 cases have been retrospectively described in the literature, and thus no incidence rates have been reported. However, a higher number of undetected cases must be assumed, especially in the pre–optical coherence tomography (OCT) era.

The etiology of AIBSES as a disease of the outer retina remains unknown. Viral illness and vaccinations have been described as potential triggers [2,3,4,5,6]. An autoimmune cause of the disease has been discussed but is considered unlikely as recurrences are almost non-existent [7]. Furthermore, the idea of choriocapillaris non-perfusion and secondary ischaemia causing dysfunction of the outer retinal layers has been debated [3].

Diagnostic tools used to characterise the features of this disease include fundoscopy, visual field examination, OCT imaging, fluorescein angiography and electrophysiology. However, because OCT can identify AIBSES as a disease of the outer retina surrounding the optic disc, it has become the gold standard. Apart from marked blind spot enlargement, other clinical and diagnostic signs are less obvious (mostly unaffected visual acuity, faint blurring of the optic disc margins, no or mild staining of the optic disc on fluorescein angiography and no P100-delay) [3,7].

The majority of the reported cases of AIBSES have been in young to middle-aged Caucasian females [1,7]. Since unilateral visual field defects and visual phenomena are also found in young adults presenting with optic neuritis, AIBSES is often misdiagnosed, which leads to unnecessary and sometimes invasive diagnostic neurological workup and therapy [8].

Other conditions presenting with blind spot enlargement are papilledema, acute zonal occult outer retinopathy (AZOOR) and multiple evanescent white dot syndrome (MEWDS), the last two of which are also diseases of the outer retina [3,7,9].

No uniform therapy regime has been established so far. The approaches range from observation, in the majority of cases, to the infrequent systemic administration of corticosteroids at different time points. According to the literature, the cure rates have been poor [5,7].

We want to emphasise that AIBSES is an ophthalmic entity that is often missed, with a high estimated number of unreported cases, especially when OCT was not available. The features that distinguish AIBSES from optic neuritis and AZOOR are debated. We present multimodal imaging of three cases, with and without early corticosteroid treatment, which in our experience, supports retinal reorganisation and restoration of the visual field. As papillary OCT is being used increasingly as a diagnostic tool in ophthalmology and neurology, this article aims to increase focus on the outer retinal layers in addition to the well-established retinal nerve fibre layer (RNFL).

## 2. Case Series

Under the assumption of optic neuritis/papillitis, a serological workup was completed prior to neuroophthalmological assessment. Infectious and autoimmune diseases were ruled out by analysing the following laboratory values: full blood count, urea and electrolytes, thyroid levels, anti-nuclear antibodies (ANAs), anti-neutrophil cytoplasmic antibodies (ANCAs), aquaporin-4 antibodies, anti-glomerular basement membrane antibodies (anti-GBM Ab), viral polymerase chain reaction (PCR) for detection of cytomegalovirus (CMV) and Epstein–Barr virus (EBV), and antibodies against Bartonella henselae, Borrelia burgdorferi, Mycoplasma pneumoniae, and Treponema pallidum. In addition, cases 1 and 2 underwent brain magnetic resonance imaging (MRI) examination and a lumbar puncture, neither of which revealed any pathological findings. In cases 1 and 3, oral azithromycin 500 mg was given preventively for three days as a broad-spectrum therapeutic.

### 2.1. Case 1

A 26-year-old Caucasian woman consulted the neuroophthalmological department complaining of acute monocular photopsia, more precisely, flickering lights on the temporal half of her left eye, which had started seven days previously. She also reported decreased vision. The referral was made by an ophthalmologist, who suspected papillitis. Apart from flu-like symptoms eight months prior to the visit, the patient’s past medical history was unremarkable.

**Figure 1:**
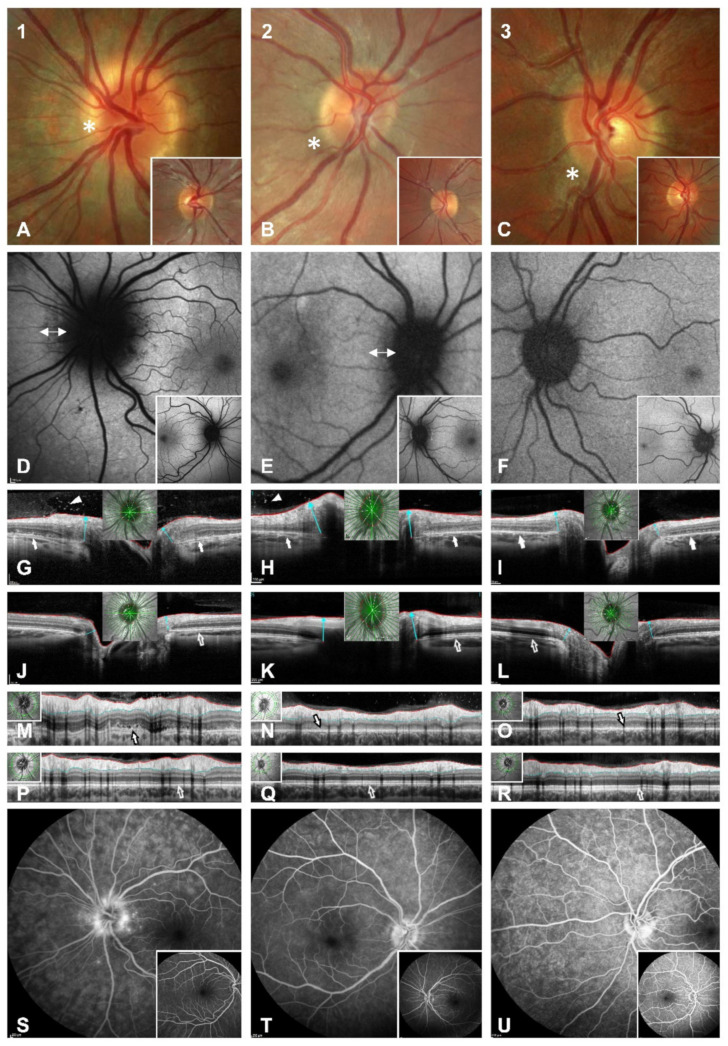
Multimodal imaging of three patients with AIBSES during the first week following the onset of symptoms. Vertical columns show, from left to right, cases 1–3, using SD-OCT (Heidelberg Engineering, Heidelberg, Germany). (**A**–**C**): Photographs of the optic disc of the affected eye, showing blurred optic disc margins and mild peripapillary colour changes (*), which were most obvious in case 1. Inlets show the contralateral eye. (**D**–**F**): Fundus autofluorescence imaging discloses circular irregular hypoautofluorescence of the peripapillary retina in cases 1 and 2, marked with arrows. Inlets show the contralateral eye. (**G**–**L**): Radial OCT cross-sections. Peripapillary irregularity and hyporeflectivity of the retinal pigment epithelium/Bruch’s membrane complex, the interdigitation zone, the outer segments of photoreceptors and the ellipsoid zone are present in all affected eyes (arrows in (**G**–**I**)). The disturbance of retinal layer anatomy is particularly apparent in comparison to the unaffected eyes (empty arrow in (**J**–**L**)). Furthermore, hyperreflectivity in the preretinal vitreous is seen in (**G**,**H**) (arrowheads). (**M**–**R**): Retinal nerve fibre layer (RNFL) analysis. Little RNFL elevation is noticeable in the affected eyes of case 1 and 2 (**M**,**N**), but not in case 3 (**O**), compared to the unaffected eyes (**P**–**R**). The impairment of the peripapillary outer retinal layers is also well-displayed (filled arrows in (**M**–**O**)). (**S**–**U**): Intravenous fluorescein angiography (FAG) depicts mild hyperfluorescence/staining of the optic nerve head edges without significant leakage. Speckled hyperfluorescent lesions are depicted in the surrounding disc area in case 1 (**S**), which are also seen in (**D**).

On examination, the patient’s best-corrected visual acuity was 20/25 in the right eye (refraction −0.25/−0.25/×118) and 20/20 in the left eye (refraction +0.50/−0.50/×42). Pupillary response was normal with no relative afferent pupillary defect (RAPD). Eye motility was normal. The red desaturation test was negative. Intraocular pressure was within the normal range. The findings of an anterior chamber slit lamp examination were physiological in accordance with age. Funduscopically, there was mild left peripapillary blurring with no haemorrhage or shadowing of the retinal vessels and a hypopigmented ring around the disc, which was not seen in the contralateral eye (Figure 1A).

**Figure 2:**
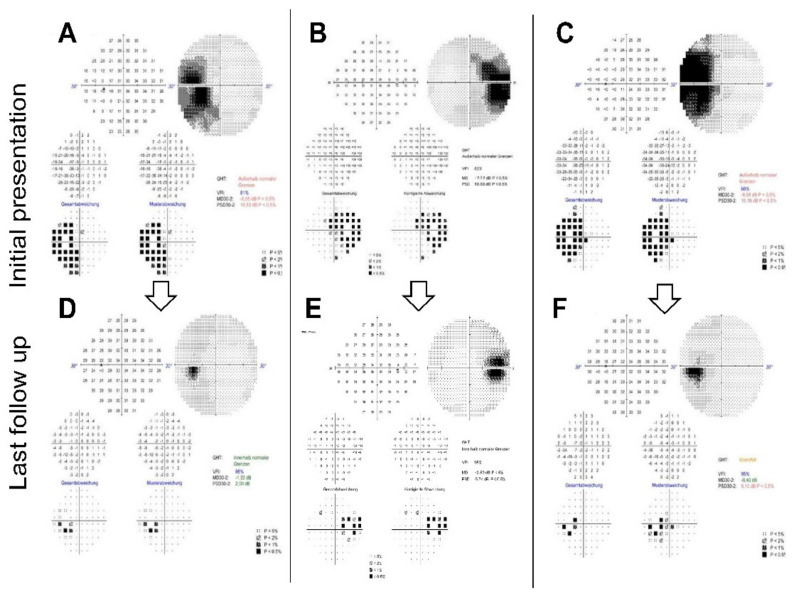
Static perimetry. Results of the 30-2 perimetry examination (Humphrey visual field analyser 3, Zeiss) of the affected eyes of cases 1–3 (from left to right) at first presentation (**A**–**C**) and at last follow-up, which was after six months in case 1 (**D**), after 12 months in case 2 (**E**) and after seven months in case 3 (**F**). Although all patients reported full recovery of their symptoms, discrete blind spot enlargement persisted in cases 2 and 3 (**E**,**F**).

A 30-2 perimetry test (Humphrey visual field analyser 3, Carl Zeiss, Oberkochen, Germany) depicted left blind spot enlargement (Figure 2A). Irregular spotted hypoautofluorescence of the peripapillary retina was found in the left eye with normal appearance in the contralateral eye (Figure 1D). Radial OCT cross-sections revealed peripapillary irregularity and hyporeflectivity of the retinal pigment epithelium/Bruch’s membrane complex, the interdigitation zone, the outer segments of photoreceptors and the ellipsoid zone (Figure 1G,J). Furthermore, hyperreflectivity was apparent in the preretinal vitreous, which was not visible by fundoscopy (Figure 1G). RNFL analysis showed very little elevation in comparison to the other eye but distinct irregularity and oedema of the outer retinal layers (Figure 1M,P). Fluorescein angiography (FAG) showed late peripapillary speckled hyperfluorescence but no leakage of the optic disc (Figure 1S). The visual evoked potentials (VEP) showed normal latencies bilaterally but borderline amplitude reduction in the left eye in comparison to the unaffected eye (data not shown).

As all the findings were highly suggestive of AIBSES, we immediately started treatment with body-weight–based oral prednisolone with an initial dose of 60 mg, which was gradually tapered over six weeks.

**Figure 3:**
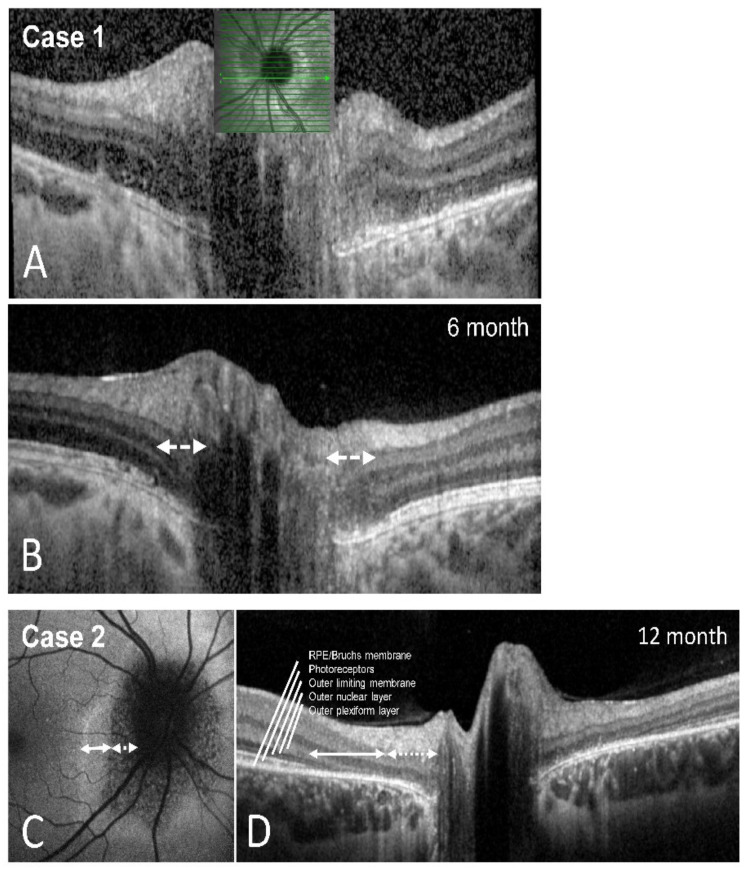
Restoration of outer retinal layer anatomy. SD-OCT scans (Heidelberg Engineering, Heidelberg, Germany). (**A**,**B**): Horizontal OCT cross sections of case 1 at first presentation (**A**) and after six months (**B**), showing restoration of the outer retinal layers in the peripapillary area except of the most proximal disc part, marked with arrows (**B**). (**C**,**D**): Autofluorescence (**C**) and OCT (**D**) of case 2 after 12 months. A hyperfluorescent and hypofluorescent area was apparent over time in the affected zone. Degeneration of the outer plexiform and outer nuclear layers (dotted arrow) represent the difference between both areas, resulting in hypofluorescence.

Perimetry at the two-month follow-up appointment showed an almost full recovery of the scotoma—the patient no longer complained of visual symptoms. The best-corrected visual acuity remained stable. A fundus examination showed left blurred disc margins and peripapillary retinal pigment epithelium (RPE) irregularity. The OCT scans of the left optic disc showed significant improvement of the outer retinal layer anatomy compared to the initial imaging. The left VEP amplitudes had recovered, and the latencies remained normal. We scheduled several follow-up appointments, which showed further improvement of the visual field and the outer retinal layers on OCT imaging. Full recovery of the blind spot enlargement and almost full restoration of the ellipsoid zone was detectable after six months (Figure 2D and Figure 3A,B).

### 2.2. Case 2

A 27-year-old female reported a five-day history of gradually worsening photophobia in conjunction with photopsia in the right eye, as well as a mild feeling of ocular pressure. The patient’s past medical history included previous episodes of migraine.

On examination, the patient’s best-corrected visual acuity was 20/20 in the right eye (refraction +0.00/−0.75/×172) and 20/20 in the left eye (refraction +0.00/−0.50/×45). Pupillary response was normal with no RAPD. Intraocular pressure and examination of the anterior chamber were unremarkable. Funduscopically, there was mild blurring of the right optic disc (Figure 1B). The right anterior vitreous showed no cells. Cells in the posterior vitreous were barely visible funduscopically. OCT, however, clearly showed preretinal hyperreflectivity (Figure 1H). A visual field examination revealed enlargement of the right blind spot (Figure 2B).

Fundus autofluorescence (FAF) showed peripapillary fuzzy hypoautofluorescence (Figure 1E). Fluorescein angiography of the right eye showed no signs of vasculitis but mild staining of the left optic nerve head margin. FAG of the left eye was unremarkable (Figure 1T). When the patient first presented, OCT showed a peripapillary disturbance of the outer retinal layers (Figure 1H,N). VEPs showed normal latencies in both eyes but a check-size dependent decrease of the right amplitudes. Responses in the multifocal electroretinogram (mfERG) were significantly reduced in the affected right eye and normal in the unaffected eye (data not shown).

Based on these findings, we diagnosed the patient with AIBSES and started treating her with oral prednisolone with an initial dose of 60 mg, which was gradually tapered over eight weeks. At the two-month follow-up appointment, the patient reported a subjective reduction of visual impairment with stable visual acuity. Perimetry showed partial but continuing improvement of the scotoma, which continued to improve during one year of follow-up. After one year, a significant but asymptomatic enlargement of the blind spot was still found (Figure 2E). OCT imaging after one year revealed persistent but narrowed peripapillary atrophy of the outer retinal layers (Figure 3C,D) and partial restoration of autofluorescence in the peripheral zone of the defect (Figure 3C).

### 2.3. Case 3

A 25-year-old Caucasian male was referred by his ophthalmologist with a suspected diagnosis of typical optic neuritis after complaining about a one-week history of flickering monocular sinistral scotoma in the temporal visual field. His past medical history was unremarkable. He had received the diphtheria-tetanus-pertussis vaccination one week prior to the initial presentation.

On examination, the patient’s best-corrected visual acuity was 20/20 in both eyes (refraction OD +0.25/−0.75/×138; OS −1.75/−0.50/×36). Red desaturation test, eye motility, intraocular pressure and examination of the anterior chamber were unremarkable in both eyes, with no signs of inflammation. Funduscopically, there was mild blurring of the left optic nerve and peripapillary brightening of the retina (Figure 1C). Perimetry showed enlargement of the left blind spot (Figure 2C). Intravenous FAG depicted mild partial hyperfluorescence of the left optic nerve head without leakage (Figure 1U). OCT imaging depicted peripapillary irregularities of the outer retinal layers, which led to the diagnosis of AIBSES (Figure 1I,O). Visual evoked potentials showed reduced amplitudes and normal latencies in the affected left eye. Responses in the mfERG were significantly reduced in the affected left eye but normal in the unaffected eye (data not shown). Since there was a significant decrease in symptoms and visual field defect size after six days, we decided against applying therapy with systemic corticosteroids.

We scheduled follow-up appointments five and seven months after the initial presentation. Perimetry over the course of seven months showed remarkable improvement without therapy, although a slight blind spot enlargement persisted (Figure 2F). Comparing OCT Bruch’s membrane opening (BMO) and RNFL scans over the course of seven months, the most recent multimodal imaging revealed partial recovery of the outer retinal layers (data not shown).

**Figure 4:**
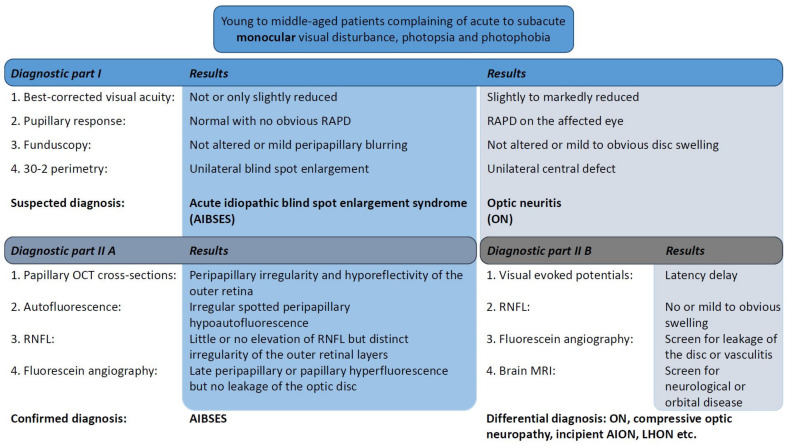
Flowchart of diagnostic workup. After the diagnostic part I (**upper part**), a sound clinical suspicion can be made to differentiate AIBSES from optic nerve diseases. The examinations of diagnostic part IIA (**left**) serve to confirm the diagnosis of AIBSES, especially with the use of papillary OCT cross-sections. Fluorescein angiography is not absolutely necessary. The diagnostic part IIB (**right**) can be used to further differentiate between optic neuritis and other retrobulbar diseases, which more often can affect young to middle-aged patients. RAPD: relative afferent pupillary defect; RNFL: retinal nerve fiber layer; MRI: magnetic resonance imaging; AION: anterior ischemic optic neuropathy; LHON: Leber hereditary optic neuropathy.

A flowchart of examinations and clinical findings is shown in Figure 4 as a guiding structure in the differential diagnosis.

## 3. Discussion

The diagnosis of AIBSES must be considered on the basis of the following cardinal findings: acute monocular blind spot enlargement in visual field examination and peripapillary outer retinal abnormalities on OCT imaging in an otherwise unremarkable fundus. The young to middle-aged patients—most of them women—complain of acute to subacute photopsia and photophobia. The condition is unknown to many ophthalmologists and neurologists and therefore is often missed.

Although optic neuritis is a disease of the optic nerve that is often associated with multiple sclerosis, and AIBSES is a disease of the outer retina, the likelihood of confusion between them is present when OCT of the outer retina is not inspected. Two of our patients were initially referred to neurology and given a diagnosis of optic neuritis. This appears reasonable at first, as patients with both diseases are generally (1) young adults, (2) female, (3) with unilateral acute visual symptoms and (4) with no, or very few, funduscopic changes. On a closer look, however, these two disease entities can be distinguished by taking into consideration the hallmarks of typical optic neuritis, which were extensively studied and described in the Optic Neuritis Treatment Trial (ONTT) [10]. The ONTT followed 457 acute optic neuritis patients for eight days following onset between 1988 and 1991 in the United States to investigate treatment with oral or intravenous corticosteroids versus placebo. The clinical profile of typical optic neuritis was summarized as follows: A total of 77.2% of the patients were women. The mean age was 31.8 years. Ocular pain on eye movement was experienced by 92.2% of the patients. The visual loss and visual field defects were severe (only 10.5% showed visual acuity of 20/20 or better in the affected eye) and objectifiable through a relative afferent pupillary defect and latency delay in VEP. The optic disc and fundus appeared normal in 64.7% of the patients or showed only mild optic disc swelling. Atypical optic neuritis must be assumed when the above criteria are not present, and one or both optic discs show significant swelling, haemorrhages or exudates in conjunction with significant and progressive visual loss without pain.

The above findings differ greatly from the clinical presentation of patients with AIBSES, who, in previous studies, did not complain of ocular pain and had normal or only slightly reduced visual acuity in the majority of cases as the central visual field was unaffected, and, therefore, no afferent defect was detectable with VEP or pupil reflex testing [1,7,11]. Instead, OCT reveals that AIBSES patients have outer retinal abnormalities in the peripapillary area, corresponding to the visual field defect, which is a prominent enlarged blind spot. When analysing the patterns of visual field loss in 229 optic neuritis patients, the 1991 Optic Neuritis Study Group found blind spot enlargement in only 2.6% of the patients [10]. Thus, the visual field defect of AIBSES patients must raise concerns about other optic nerve diseases, which typically present with central or nerve fibre bundle visual field defects [12]. None of our patients reported pain, nor was there any reduction in best corrected visual acuity, and the VEP latencies were within normal limits. In a study of 27 patients with AIBSES, 23 complained of positive visual phenomena and 16 patients presented with a visual acuity of 20/20, 10 with a visual acuity between 20/25 and 20/50 and only one with a visual acuity of 20/200 [7].

In the fewer than 100 published cases of AIBSES [1,7,11,13,14], about two-thirds of the patients are women. Although most AIBSES patients are young adults, an age range of 16 to 63 years has been reported [1,7,11,13,14,15]. Unilateral courses are the rule. Recurrence of the disease cannot be ruled out but is highly uncommon. Six of the 27 cases described by Volpe suffered a recurrence between one and 15 years after the first occurrence. In two cases, the contralateral eye was affected [7].

Although no optic disc swelling was found in our patients, blurring of the optic disc margins was seen, and discrimination between possible diagnoses by fundoscopy alone was unfeasible. Blurred margins of the optic disc have been previously described as a common funduscopic finding representing peripapillary retinal damage [7], although other authors have reported no abnormalities of the optic disc [7,16]. Vitritis was present in only three of the 27 cases in Volpe et al.’s study, the largest AIBSES cohort collected to date. Vitreal hyperreflectivity was found adjacent to the inner retinal layers in two out of the three cases presented here, depicted in OCT images (Figure 1G,H). Due to this location, there was no funduscopic evidence of cells in the anterior vitreous, which is accessible by indirect fundoscopy. Fluorescein angiography revealed late staining of the optic nerve head edges in our three patients and mild peripapillary leakage in one case (Figure 1S,U). This finding was present in almost 50% of the 27 patients described previously [7].

The gold standard for diagnosing AIBSES nowadays, however, is a papillary SD-OCT. Common findings include microstructural irregularities and loss of the ellipsoid zone and outer nuclear layers of the retina [4,17,18]. Some authors have described cases in which recovery of the ellipsoid zone on OCT imaging, formerly known as the inner and outer segments of retinal photoreceptors, corresponded with visual improvement, which is consistent with our findings [17,18]. Over the observation period of our cases, recovery of the irregular outer retina was found (Figure 3). Therefore, we highlight the importance of SD-OCT for the diagnosis of AIBSES and for follow-up.

In the pre-OCT era, the use of ERG to detect abnormalities of the retinal photoreceptors was suggested for diagnosing patients with AIBSES [1,5,7,19]. Multifocal electroretinogram amplitudes were abnormally reduced in both of our patients who had ERG, consistent with the results of previous studies, in which eight of nine patients showed abnormal nasal parafoveal focal ERG results [7]. In their retrospective analysis of 22 patients, Watzke et al. showed that multifocal ERG results revealed more extensive and even bilateral retinal damage than perimetric results and clinical examination would have suggested [13]. Interestingly, Piri et al. also describe a case with reduced perifoveal mfERG responses in the asymptomatic contralateral eye [17]. This was not a finding in our patients, where we found changes only in the symptomatic eye without evidence of changes to the unaffected eye. In summary, multifocal ERG can be considered a supporting diagnostic tool for characterising outer retinal function in AIBSE patients but is dispensable in cases with an unambiguous OCT finding.

No general agreement has been reached regarding the exact classification of AIBSES in relation to other disease entities affecting the outer retina or choriocapillaris, especially AZOOR and MEWDS [4]. Whether AIBSES is an independent disease or just a facet of AZOOR [7,20] is still being debated. This is reasonable, as AIBSES and AZOOR show some similarities. In both disease entities, changes at the level of the outer retinal bands on OCT seem to lead to photopsia and visual field loss on perimetry, but, interestingly, without decreasing visual acuity significantly [2]. On SD-OCT, AZOOR patients show changes at the photoreceptor level—mainly irregularities of the ellipsoidal zone, drusenoid deposits and atrophy of the photoreceptors and retinal pigment epithelium (Mrejen et al., 2014). Young women are particularly affected—the average age of AZOOR patients is 30 years [2]. Further similarities include the mostly inconspicuous funduscopic findings at the beginning of the disease and the occasional finding of cells in the vitreous body. However, after reviewing the literature and based on our findings, we argue in favour of classifying AZOOR and AIBSES as different entities. AZOOR is often described as bilateral (in nine of 13 patients described by Gass [2]), and ERG changes were measureable in both eyes in 50% of patients, some of whom were clinically asymptomatic [2]. AIBSES however, is a unilateral disease. While in AIBSES the enlargement of the blind spot is the pathological hallmark resulting from the one peripapillary focus, an exclusive enlargement of the blind spot is rarely found in AZOOR patients—only in 19% of the published cases, which mostly show multifocal visual field defects [19]. Of the 51 patients reported in the largest published AZOOR cohort to date, recurrence of the disease with at least one episode occurred in 16 cases. The median time to recurrence was 39 months [19]. Documented recurrences of AIBSES are rare, as reported above. Finally, another distinctive feature seems to be the poor recovery of photopsias and visual field defects in AZOOR patients, in whom depigmentation and atrophic changes of the retinal pigment epithelium developed and correlated with the persisting visual field defects [19]. In contrast, our AIBSES patients showed a good regression of their symptoms, with and without corticosteroids, as well as a significant improvement of the visual field defect over time.

While the clinical symptoms of MEWDS and AIBSES are often similar (unilaterality; spontaneous occurrence of photopsia; visual field defects), diagnostic differences can help to distinguish the two entities. The funduscopically faint white dots in the mid-periphery, which give MEWDS its name, were not present in our patients, nor have they been described for AIBSES patients. The fluorescence angiography of our patients showed hyperfluorescence exclusively around or on the optic disc. Patchy areas of late hyperfluorescence in the periphery, as described for MEWDS, were not found, nor were peripheral changes in fundus autofluorescence. MEWDS is known to be a self-limiting disease while spontaneous recovery in AIBSES has not yet been reported in the majority of cases.

The etiology of AIBSES remains unclear. For both AIBSES and AZOOR, viral infections, autoimmune diseases and vaccinations have been found to be potential triggers [2,3,4,5,21]. Genetic and hormonal factors are also possible, with the clear predominance of female patients [7]. Cimino et al. suggest that choriocapillaris non-perfusion and secondary ischemia may cause dysfunction of the outer retinal layers, and this may be the mechanism behind primary inflammatory choriocapillaropathies (PICCP), including AIBSES and MEWDS [3]. However, none of our patients reported a recent history of a viral infection or disease, while only one patient (case 3) reported recent vaccination. However, a careful history of COVID-19 disease and vaccination showed no causal relationship with the beginning of visual symptoms in this patient. In our opinion, an autoimmune/parainfectious inflammatory etiology can be hypothesised, as vitreous cellular hyperreflectivity is often present preretinally but is visible only in the OCT. We can only speculate about the potential impact of steroid treatment on recovery to support this idea.

Currently, no established therapeutic regime exists to treat AIBSES. The use of systemic steroids has been reported in single case descriptions. The reported results vary from spontaneous improvement of signs and symptoms without intervention within six to 10 weeks to the use of systemic immunosuppressive drugs [3,4,5]. There are no references to potential therapies in either the earliest description of AIBSES [1] or in the largest collection of 27 patients [7]. The majority of individual case descriptions and small case series, however, report no or only partial regression of the visual field defect [1,5,7,18]. We saw visual field improvement in all our prospectively followed patients, two of whom received treatment with systemic steroids and one of whom received no treatment but recovered spontaneously very quickly. With a relatively low spectrum of side-effects and an impressive reduction in visual field defects and irregularities of the outer retina on OCT in both of our patients treated with systemic steroids, we currently consider the administration of weight-adapted corticosteroids for some weeks to be advisable for the treatment of AIBSES. Further controlled studies are needed to draw stronger conclusions about the impact of timing and dosing.

## 4. Conclusions

To the best of our best knowledge, fewer than 100 cases of AIBSES have been published, and the accurate classification of disease entities affecting the outer retina continues to pose difficulties. However, a higher number of undetected cases must be assumed, especially in the pre-OCT era. A careful history taking, and unprejudiced ophthalmological workup, helps in diagnosing AIBSES in young adults in whom unilateral acute blind spot enlargement is identified in visual field examination. Although the etiology is still unclear, AIBSES can be accurately diagnosed with peripapillary OCT, perimetry and a lack of a distinct relative afferent pupillary defect. An autoimmune/parainfectious inflammatory etiology seems possible as preretinal vitreous cellular hyperreflectivity is often present. Early treatment with corticosteroids may support outer retinal reorganisation and restoration of the visual field; precise dosage, administration and duration of treatment should be addressed in detail in a prospective study. The course of our prospectively followed patients raises doubts about the often-stated view that AIBSES is a disease with a poor prognosis. AIBSES is a noteworthy ophthalmologic condition that can be clearly distinguished from optic neuritis. Neurologists should be suspicious if referred patients show unilateral blind spot enlargement, which is a very rare finding in optic neuritis. As there is an increasing use of papillary OCT diagnostic in ophthalmology and neurology, this article aims to increase focus on the outer retinal layers in addition to the familiar RNFL.

## Data Availability

Not applicable.
